# Tricuspid Valve Infective Endocarditis

**DOI:** 10.7759/cureus.114058

**Published:** 2026-08-06

**Authors:** Zahraa M Al Dujaili, Asl Abu-Nayla, Ahila Abu-Nayla, Abdulameer Abu Nailah

**Affiliations:** 1 Hematology, Portsmouth Hospitals University NHS Trust, Portsmouth, GBR; 2 General Surgery, American Hospital Dubai, Dubai, ARE; 3 Internal Medicine, Prime Hospital, Dubai, ARE; 4 Rheumatology, Canadian Specialist Hospital, Dubai, ARE

**Keywords:** chest pain, severe sepsis, staphylococcus aureus infection, tricuspid valve endocarditis, tricuspid valve regurgitation

## Abstract

*Staphylococcus aureus* endocarditis is a severe and life-threatening infection that continues to pose a significant challenge in cardiovascular medicine. Despite advances in healthcare, its management remains complex due to its aggressive nature and potential complications.

Although infective endocarditis (IE) predominantly affects the left side of the heart, right-sided IE can also occur, often linked to intravenous drug use, medical device implantation, and vascular access for dialysis. Notably, many patients with native valve *S. aureus* endocarditis have no prior structural heart disease, suggesting that inflammation may play a critical role in its development.

Early diagnosis is crucial and relies on comprehensive clinical evaluation, blood cultures, and echocardiography. Empiric antibiotic therapy should provide broad coverage against common pathogens, with adjustments based on culture results. In certain cases, surgical intervention is required, particularly when complications such as large vegetations, persistent infection, or severe valve dysfunction arise.

A multidisciplinary approach involving infectious disease specialists, cardiologists, and cardiothoracic surgeons is essential for optimizing patient outcomes. Timely intervention, individualized treatment strategies, and coordinated care can significantly reduce the risk of complications and improve prognosis.

## Introduction

*Staphylococcus aureus*, a bacterium commonly found on the skin and mucous membranes, can enter the bloodstream through various means, such as skin wounds, surgical procedures, or intravenous catheters. Once in the bloodstream, the bacteria can attach to damaged areas of the endocardial lining or heart valves, facilitated by factors such as platelets, fibrin, and other molecules. This triggers an inflammatory response, leading to the formation of vegetations. These vegetations consist of bacteria, blood cells, fibrin, and other substances. The vegetations can grow in size and cause mechanical stress on the valve leaflets, leading to microscopic damage.

Fragments of vegetations or emboli can lodge in smaller blood vessels, causing blockages and potentially leading to damage in other organs, such as the lungs, brain, or kidneys. This can result in complications such as septic emboli, stroke, or kidney damage. The presence of *S. aureus* sepsis and infective endocarditis (IE) can lead to a range of clinical manifestations. Patients may experience fever, chills, fatigue, night sweats, and various cardiac symptoms such as murmurs or changes in heart sounds found on examination. 

Empirical antibiotic therapy is initiated promptly after obtaining blood cultures to identify the causative pathogen, including whether it is methicillin-sensitive *S. aureus* or methicillin-resistant *S. aureus*. The duration of antibiotic therapy can vary based on factors such as the type of bacteria, the extent of infection, and the patient's response to treatment. Typically, treatment courses are several weeks long, and a portion of the treatment may be administered intravenously while transitioning to oral antibiotics as the patient improves.

Surgery may be necessary in cases of severe IE, particularly when there is valve dysfunction, heart failure, or uncontrolled infection. Surgical options include valve repair or replacement, removal of infected tissue (debridement), and drainage of abscesses. 

Patients with *S. aureus* sepsis and IE often require supportive care to manage the systemic effects of infection. This includes maintaining fluid and electrolyte balance, treating any complications (such as septic emboli), and addressing hemodynamic instability. Follow-up blood cultures, imaging studies, and clinical assessments help gauge the response to treatment and identify any potential complications. 

## Case presentation

A 41-year-old Emirati male patient, a smoker and known carrier of hepatitis C for 15 years, was transported by ambulance due to severe chest pain. Documentation regarding intravenous drug use (IVDU), recent dental infection or dental procedures, and HIV status was not available in the reviewed hospital records. He has a previous history of inguinal hernia surgery. He had experienced left-sided chest pain radiating to his back for the past day, with a pain score of 7-8/10. In addition, he had been running a fever for two days, accompanied by a cough and shortness of breath. Although he had received medication at a clinic two days ago, his symptoms had not improved. The patient denied any vomiting, abdominal pain, dizziness, headache, or gait disturbances.

Upon arrival at the ER, his vital signs were as follows: temperature: 38.2°C, heart rate: 110/minute (regular), respiratory rate: 24/minute, SpO_2_: 92% on room air, and SpO_2_: 98% with 3 L/min of oxygen via nasal cannula. His blood pressure was 92/56 mmHg. During the examination, he was conscious, oriented, and sweating profusely. He exhibited jaundice and mild pallor. His chest revealed harsh vesicular breath sounds, with equal air entry bilaterally and reduced sounds at the bases, along with a few basal crepitations. Heart sounds S1 and S2 were clear without any murmurs, peripheral pulsations were palpable, and there was no peripheral edema. His abdomen was soft, with mild generalized tenderness upon deep palpation. Urticarial rashes were noted on his skin, along with scattered pustules displaying red margins.

**Figure 1:**
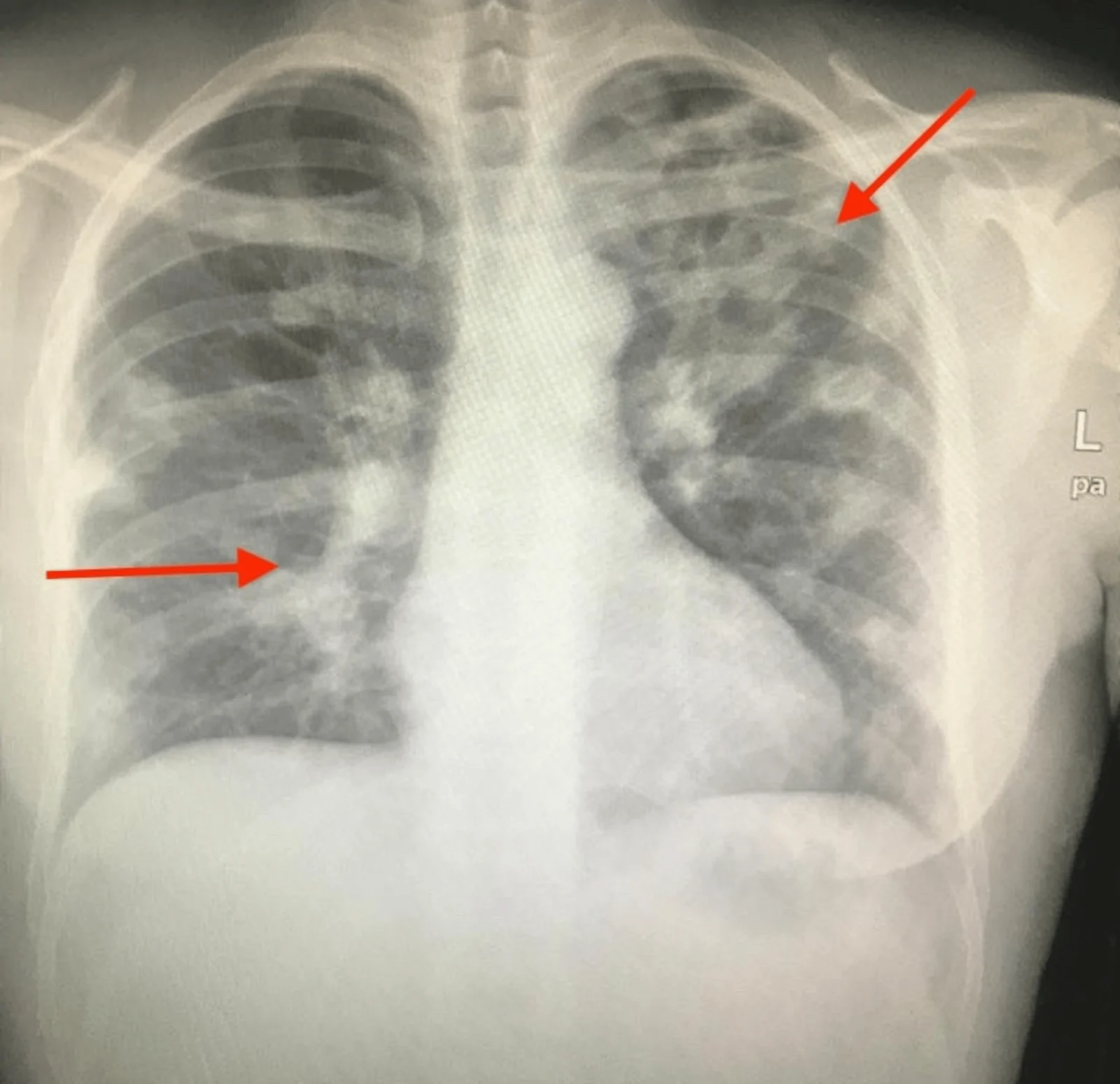
The chest X-ray shows bilateral bronchovascular markings, increased haziness, an obliterated left costophrenic angle, and no pneumothorax.

The chest X-ray was taken in a posteroanterior view that showed bilateral bronchovascular markings, increased haziness, an obliterated left costophrenic angle, and no pneumothorax (Figure 1). The patient was started on IV fluids and admitted to the high dependency unit under the care of an internal medicine specialist for a full sepsis workup and management.

**Figure 2:**
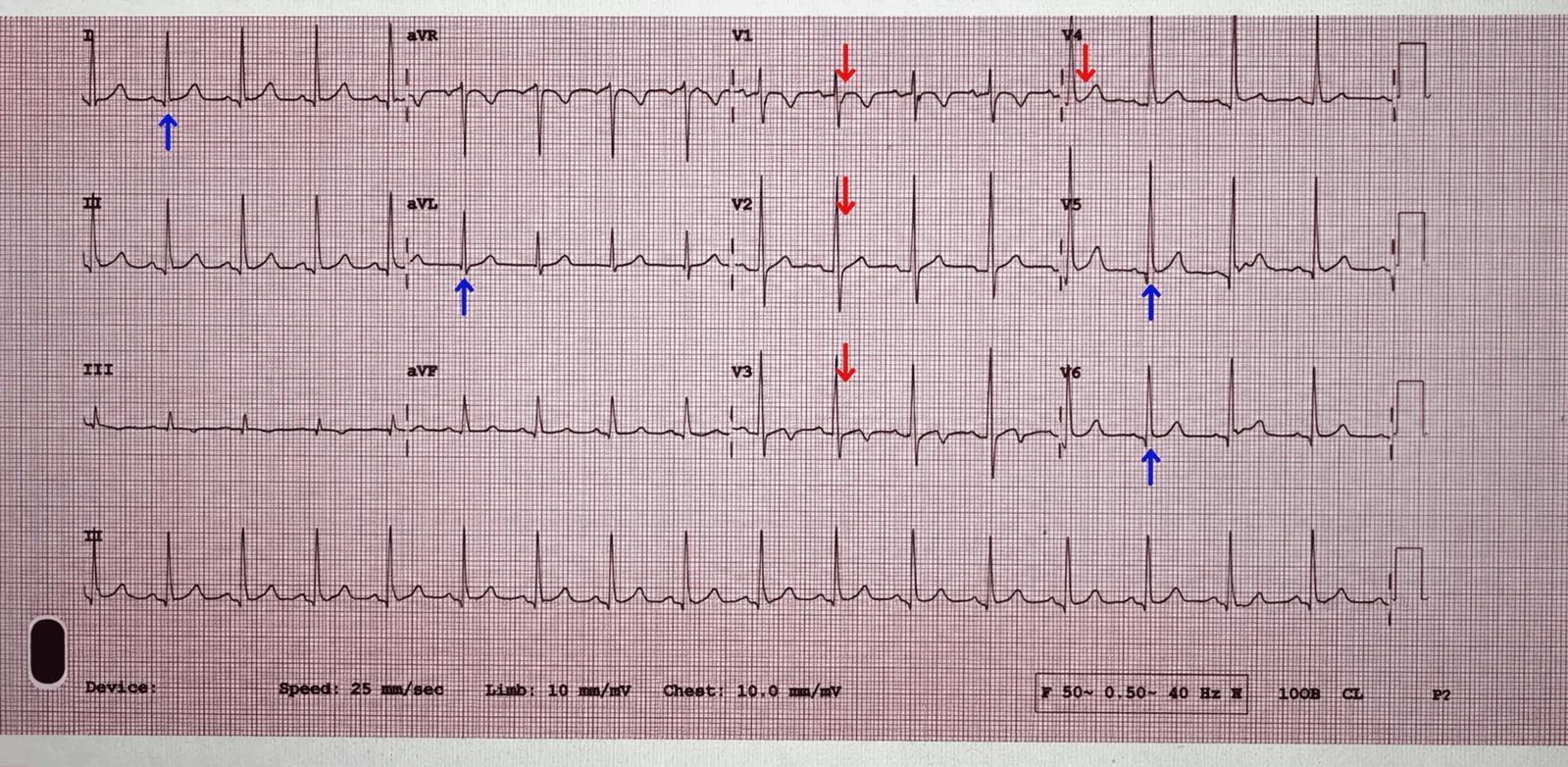
ECG of the patient revealed a heart rate of 106 beats per minute, left ventricular hypertrophy, T-wave inversion in leads VI-V4, and a prolonged QTc interval of 451 milliseconds. Red arrows indicate T-wave inversions in leads V1-V4. Blue arrows indicate representative high-voltage QRS complexes consistent with left ventricular hypertrophy.

Figure 2 presents the ECG revealing a heart rate of 106 beats per minute, left ventricular hypertrophy, T-wave inversion in leads VI-V4, and a prolonged QTc interval of 451 milliseconds.

The cardiologist's opinion was sought, ruling out an acute cardiac event and advising to hold antiplatelets. Table 1 shows the blood investigations that were conducted. The increased bilirubin level was most likely due to sepsis, and since the liver enzymes were normal, the gastroenterologist suggested continuing supportive therapy and follow-up in the outpatient clinic after discharge. The rapid COVID-19 test returned a negative result.

**Table 1:** Blood investigations of the patient along with the normal reference values Reference values in the table are for a specific gender and age group.

Parameter	Result or value	Normal value
Blood investigations		
White blood cell (WBC) count	17.56 x 10^9^/L	4.0-10.0 x 10^9^/L
Hemoglobin (Hb)	10.7 g/dL	14.0-17.5 g/dL
Neutrophils	81.70%	40%-80%
Platelet count	175 x 10^9^/L	150-450 x 10^9^/L
C-reactive protein (CRP)	322 mg/L	≤6 mg/L
Creatinine	1.15 mg/dL	0.6-1.3 mg/dL
Blood urea nitrogen (BUN)	33.7 mg/dL	7-20 mg/dL
Random blood sugar (RBS)	180 mg/dL	70-140 mg/dL
Electrolyte levels		
Sodium (Na)	130 mmol/L	135-145 mmol/L
Potassium (K)	3.54 mmol/L	3.5-5.0 mmol/L
Bicarbonate (HCO_^3^_)	27.3 mmol/L	22-30 mmol/L
Cardiac biomarkers		
Troponin T	9.07 ng/L	≤14 ng/L
Creatine kinase MB (CK-MB)	0.629 ng/mL	0.0-3.6 ng/mL
Other blood parameters		
Total protein	6.05 g/dL	6.4-8.3 g/dL
Total bilirubin	5.442 mg/dL	0.3-1.2 mg/dL (total)
Direct bilirubin	4.1 mg/dL	0.1-0.3 mg/dL (direct)
Liver enzymes		
Alkaline phosphatase (ALP)	104 U/L	44-147 U/L
Albumin	2.46 g/dL	3.5-5.0 g/dL
Serum glutamate-pyruvate transaminase (SGPT)	53 U/L	7-56 U/L
Serum glutamic oxaloacetic transaminase (SGOT)	25 U/L	8-40 U/L
Pancreatic enzymes		
Amylase	48 U/L	25-125 U/L
Lipase	20 U/L	0-160 U/L

Echocardiography showed normal cardiac chamber size, no regional wall motional abnormality, normal left ventricular systolic and diastolic function (ejection fraction: 60%-65%), trivial mitral regurgitation, and a large vegetation measuring 1.3 × 1.0 cm attached to the septal leaflet with mild-moderate eccentric tricuspid regurgitation (TR). A small vegetation was also noted in the anterior mitral valve leaflet with trivial regurgitation. Pulmonary artery pressure was normal, as was right ventricular size and function.

**Figure 3:**
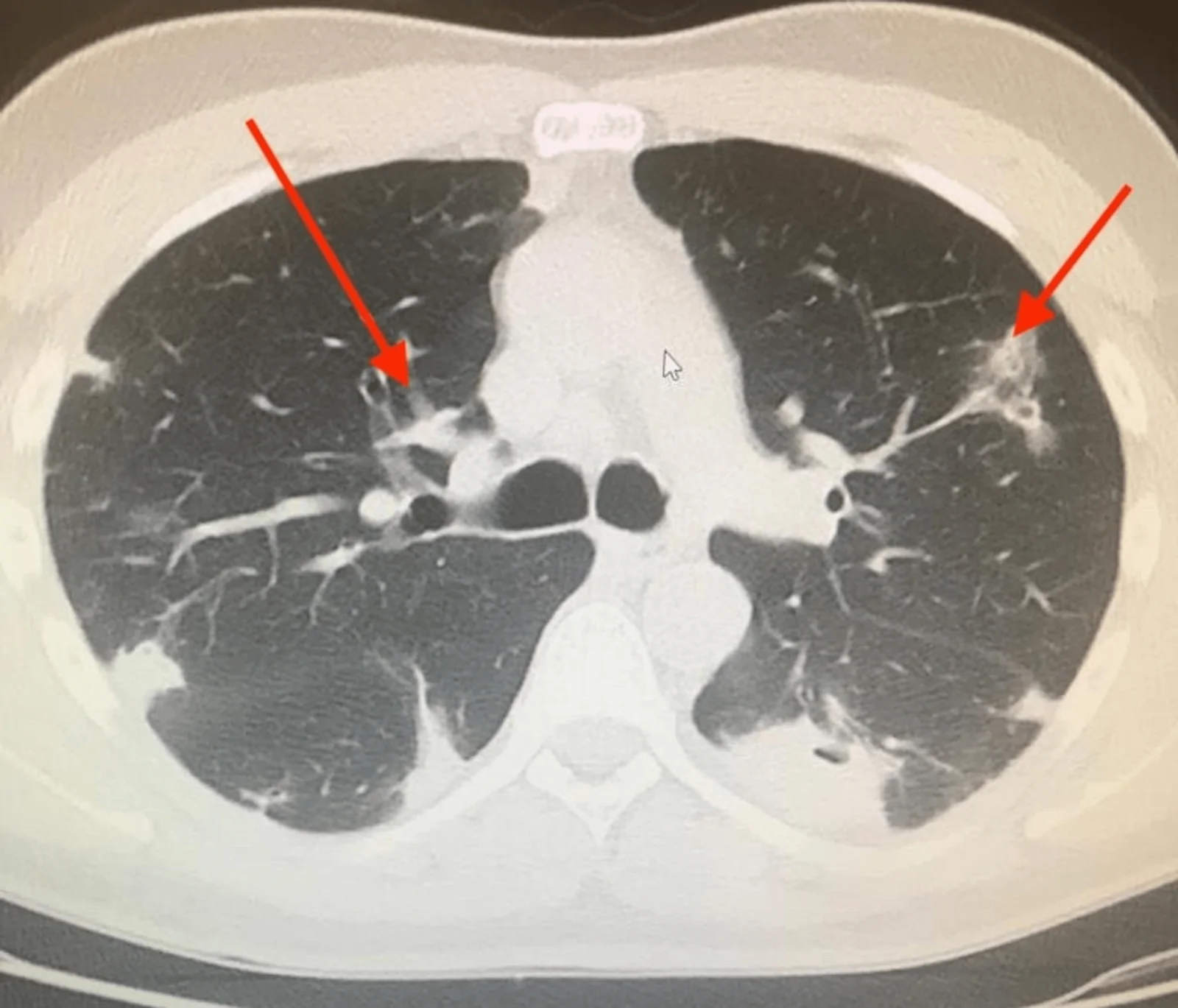
CT scan shows multifocal areas of cavitatory consolidations scattered in both lungs.

CT chest (Figure 3) was performed and showed multifocal areas of cavitatory consolidations scattered in both lungs, predominantly in the peripheral and subpleural locations, bilateral pleural effusion, minimal pericardial effusion, and mediastinal adenopathy. Findings represent septic emboli, and the patient was also found to have positive *Mycoplasma pneumoniae* IgM antibodies.

Blood and sputum cultures showed growth of *S. aureus* sensitive to the ongoing antibiotics. Table 2 presents the medications administered to the patient, along with diclofenac patch twice daily for 5 days and linezolid 600 mg twice daily for 10 days at discharge. 

**Table 2:** Medications given to the patient during hospitalization

Medication	Dose
Meropenem 1 g	Three times daily for 14 days
Levofloxacin 500 mg	Once daily for 14 days
Fluconazole 150 mg	Once daily for 10 days
Vancomycin 1 g	Three times daily for 5 days
Clexane 60 mg	Twice daily for 5 days
Xarelto 20 mg	Once daily for 4 days
Pantozol 40 mg	Once daily for 14 days
Enterogermina	Twice daily for 7 days
Paracetamol 1 g	Three times daily for 5 days

There was also moderate growth of Candida in sputum for which IV antifungal therapy was initiated. Due to poor oral intake, internal medicine team started protein supplementation in view of poor nutrition and hypoalbuminemia. The patient was also kept on anticoagulants. He became hemodynamically stable, maintained saturation on room air, and became afebrile.

## Discussion

*S. aureus* endocarditis is associated with high mortality, with reported mortality rates of approximately 20%-40% [1]. Despite advancements in preventing and treating cardiovascular ailments, the incidence and lethality of *S. aureus* endocarditis have surged in recent years [2]. 

IE represents a severe medical condition associated with elevated morbidity and mortality, primarily impacting the left side of the heart. Right-sided IE (RSIE) is relatively rare, accounting for only 5%-10% of IE cases [3].

In the realm of RSIE, the majority of cases involve the tricuspid valve (TV), with pulmonic valve involvement being less common. RSIE exhibits strong associations with IVDU, medical device implantation, and vascular access for dialysis [4]. Additionally, individuals with uncorrected congenital heart disease face an increased risk of RSIE.

Pathogens gain access to the bloodstream through various routes, such as intravenous catheters, injection drug use, or dental sources. These pathogens adhere to areas of abnormal cardiac valve surfaces. Some pathogens, notably* S. aureus*, manage to gain intracellular access to the valve endothelium. The infected vegetative growths are formed as the proliferating organisms become enveloped within a protective matrix of serum molecules. These vegetative particles can detach and disseminate, leading to complications such as ischemic stroke, mycotic aneurysms, infarctions, or abscesses in remote locations [5].

Previously, it was believed that heart valve damage predisposed individuals to endocarditis, especially in patients with congenital or rheumatic heart disease [6]. However, approximately 40% of patients with native valve *S. aureus* endocarditis had structurally normal heart valves before infection, suggesting that inflammation, rather than valve damage, might be the trigger [7]. It has been hypothesized that endocarditis, in such cases, may result from cardiac valve inflammation, marked by widespread endothelial cell activation.

Empirical antibiotic therapy for tricuspid valve infective endocarditis (TVIE) should encompass common pathogens, including Staphylococcus and Streptococcus. Initially, vancomycin is a preferred choice, but treatment should be customized based on blood culture results [8]. Surgical intervention is contemplated in specific scenarios, such as large vegetations, persistent bacteremia, and severe TR accompanied by right-sided heart failure [9].

Routine laboratory values in IE often lack specificity. A basic workup should be conducted to identify potential abnormalities such as leukocytosis, elevated erythrocyte sedimentation rate (ESR), elevated C-reactive protein (CRP), normocytic anemia, or urinalysis indicating hematuria, proteinuria, or pyuria. Electrocardiography may reveal conduction anomalies such as heart block [10]. Chest radiographs are essential for evaluating septic emboli or infiltrates. A CT scan of the abdomen aids in evaluating metastatic emboli, encompassing splenic or renal infarctions. Clinicians should maintain a low threshold for identifying these potential foci. Blood cultures must be obtained in all individuals suspected of having TVIE, with a minimum of three sets collected from different sites. Echocardiography is imperative for all individuals with suspected TVIE. Transthoracic echocardiogram (TTE) serves as the initial test, offering high specificity. Transesophageal echocardiography (TEE) should be pursued if TTE results are negative, but clinical suspicion for IE remains high. TEE may also be employed in cases of significant valve regurgitation that require further assessment before surgery [11].

The indications and timing of surgery in RSIE remain somewhat ambiguous but should be contemplated in the presence of any of the following: TV vegetations exceeding 20 mm in size, coupled with recurrent septic pulmonary emboli, with or without concomitant right heart failure. IE is attributed to microorganisms that are challenging to eradicate (e.g., fungi) or bacteremia persisting for at least seven days (e.g., *S. aureus* and *Pseudomonas aeruginosa*), despite appropriate antimicrobial therapy. Right heart failure stemming from severe TR, with limited response to diuretic therapy [12].

With growing experience in managing TVIE, surgical intervention demonstrates in-hospital mortality rates comparable to or lower than those of antibiotic therapy alone. The timing of surgery in RSIE remains less well-defined than in left-sided IE. Early intervention helps prevent further septic pulmonary embolism and additional damage to the TV leaflet tissue, increasing the likelihood of a successful repair. Early surgical consideration for TVIE is warranted in cases of concomitant left-sided IE, atrial septal defect, infected indwelling catheters or pacing leads, and prosthetic valve endocarditis [13].

The fundamental principles of surgery for TVIE encompass radical debridement of vegetations and infected tissue, alongside valve repair whenever feasible. In instances where the valve is significantly damaged and deemed non-repairable, replacement becomes imperative [14]. If pulmonary pressures and vascular resistance are elevated, and the pulmonary vascular resistance is low-normal, excising the valve without replacement may serve as a temporary solution [15]. Fortunately, TV repair can be achieved in most cases, even those with extensive valve destruction, employing various techniques [16]. These techniques may involve autologous pericardial patch augmentation of the damaged leaflets, implantation of an annuloplasty ring, and the use of expanded polytetrafluoroethylene neo-chords as needed.

Upon completing antibiotic therapy, a TTE should be performed to establish a new baseline reference for assessing valve appearance, valvular regurgitation severity, and left ventricular function. Furthermore, laboratory tests, including WBC count, ESR, and CRP, should be performed to serve as a new baseline reference.

Patients who have successfully recovered from IE face a heightened risk of recurrent IE. Therefore, all individuals with a prior IE should receive education regarding possible symptoms and signs of IE. They should also be aware of the importance of prompt medical care if these symptoms occur, including the necessity of obtaining blood cultures before initiating antibiotic treatment. Clinical evaluations, including echocardiography, should be performed if signs or symptoms of IE recurrence or worsening valve disease become evident.

Considering the potentially life-threatening nature of endocarditis, patients presenting with complications should be promptly transferred to specialized centers equipped with comprehensive cardiology and cardiothoracic surgical expertise. Timely surgical intervention is warranted in cases of heart failure, uncontrolled infection, or the need to prevent embolization or neurological complications.

## Conclusions

*S. aureus* endocarditis pathogenesis is complex, and prevention relies mainly on risk reduction, early recognition and treatment of bacteremia, appropriate infection-control measures, and guideline-based antibiotic prophylaxis only for selected high-risk patients undergoing relevant procedures; currently, there is no routinely licensed vaccine for the prevention of *S. aureus* IE. This case highlights the importance of early diagnosis and appropriate management through a multidisciplinary approach involving infectious disease specialists, cardiologists, and cardiothoracic surgeons. While endocarditis is often associated with pre-existing valve damage, a substantial number of cases occur in patients with structurally normal valves, suggesting that inflammation may play a key role in disease onset.

Accurate and timely diagnosis using blood cultures, echocardiography, and clinical evaluation is crucial for optimizing outcomes. Surgical intervention should be considered in cases of large vegetations, persistent bacteremia, or severe valve dysfunction. Additionally, systemic complications such as septic emboli require comprehensive supportive care. The patient’s improvement with tailored therapy underscores the value of individualized treatment strategies. In summary, managing *S. aureus* endocarditis requires early intervention, appropriate antibiotic therapy, and, when necessary, timely surgical consideration -- all within a collaborative, multidisciplinary framework to reduce complications and improve prognosis.
